# A New Blow-Pipe

**Published:** 1878-08

**Authors:** 


*A New Blow-pipe.*—In the days of gold and silver plate, of backings and soldering, the skillful use of the blow-pipe was a *sine qua non* to successful dental mechanism. He who could not blow well, failed in the very point where success was most essential. Now-a-days, when plastic materials have so nearly taken the place of the metals, the use of the blow-pipe is almost obsolete, few of our young men having more than a theoretical knowledge of its uses; and still there is occasional soldering to do, if it only be to mend the work of some old dentist, which after doing good service for twenty or twenty five years is so good still as to only require the replacement of a tooth, or the resoldering of a backing. And it may be too, that, a fair representation to our patients of the utility and durability of a metallic piece may result in the adoption of gold by some one whose prejudices are against it by misrepresentation or ignorance. The best appliances should be in the hands of the dentist, and of these the old fashioned mouth blow-pipe is most convenient and available.

Lately Mr. Thomas Fletcher has made an improvement to the old-fashioned mouth blowpipe which is simple, cheap and effective, and adds wonderfully to its heating power. The change or addition consists simply of a coil or series of coils made in the pipe just before reaching the tip, by means of which the air from the mouth is highly heated before issuing from the orifice of the pipe. The arrangement adds greatly to the powers of this instrument and makes it much more effective. The superheating of the air from the mouth dries it thoroughly, rendering a bulb for catching the moisture from the breath superfluous. To all who have even occasional use for the blowpipe, we can confidently recommend this instrument. H.

